# “I think it is woven through me, and sadly that means it is woven through our family life”: the experiences and support needs of mothers with eating disorders

**DOI:** 10.1186/s40337-023-00868-y

**Published:** 2023-08-29

**Authors:** Laura Chapman, Sam Cartwright-Hatton, Kathryn J. Lester

**Affiliations:** School of Psychology, University of Sussex, Falmer, Brighton, BN1 9QH UK

**Keywords:** Eating disorders, Parental mental health, Parenting, Qualitative, Thematic analysis

## Abstract

**Background:**

Eating disorders may disrupt parenting, and there is evidence to suggest that the children of parents with eating disorders are at greater risk for the development of eating disorders themselves. This study sought to broaden and extend current understandings of the lived experiences of mothers who have eating disorders.

**Method:**

A qualitative study using thematic analysis was conducted. Eighteen mothers living in the UK, USA, and Australia took part in semi-structured online interviews. Participants were mothers to at least one child aged two years or older, had received a lifetime diagnosis of one or more eating disorders, and reported experiencing symptoms since becoming a parent.

**Results:**

Data were analysed using an inductive approach to reflexive thematic analysis. Four major themes, each with subthemes, were identified. These were: parenthood as a double-edged sword; the eating disorder impacts on parenting; blame and burden; and seeking support.

**Conclusions:**

The lived experiences of mothers indicate a complex relationship between eating disorders and parenthood. While parenting can impact eating disorders, eating disorders can also impact parenting, in a range of ways that extend beyond the domains of food, eating and the body. There is a pressing need for the development of specialised, non-judgemental support options for parents with eating disorders and their families.

**Supplementary Information:**

The online version contains supplementary material available at 10.1186/s40337-023-00868-y.

## Abstract (plain language summary)

Many adults with eating disorders are parents. Previous research studies suggest that these parents may experience particular challenges in relation to parenting, and that their children may be at greater risk of developing eating disorders themselves. We interviewed eighteen mothers who had at some point in their life been diagnosed with an eating disorder, and had experienced symptoms since becoming a parent. Interview data were analysed and four themes were identified to describe the lived experiences of mothers who have eating disorders. These themes, which we refer to as ‘parenthood as a double-edged sword’, ‘the eating disorder impacts on parenting’, ‘blame and burden’, and ‘seeking support’ suggest a complex relationship between eating disorders and being a parent. Parenting can impact eating disorders, and eating disorders can also impact parenting. The mothers who participated in our study also identified unique challenges when seeking treatment, and there is a need for support options to be developed specifically for parents with eating disorders, and for their families.

**Supplementary Information:**

The online version contains supplementary material available at 10.1186/s40337-023-00868-y.

## Background

Eating disorders are widespread, with an estimated lifetime prevalence of approximately 8.4% for women and 2.2% for men [1]. Whilst onset typically occurs during adolescence and young adulthood [2], these highly stigmatized illnesses [3] can take a chronic course [4], and a significant proportion of adults accessing eating disorder treatment are parents [5]. As Stitt & Reupert highlight, eating disorders can have severe adverse effects on individuals that can also extend to the individual’s family unit [6], and there is evidence to suggest that the children of parents who have an eating disorder are at an increased risk of a range of both feeding and psychological difficulties [7], including the development of eating disorders themselves [8]. Despite this, parents with eating disorders tend to be a neglected group, both clinically and in research [6].

Both genetic and non-genetic factors are implicated in the intergenerational transmission of mental health difficulties [9], and a substantial genetic component to the heritability of eating disorders has been identified (see Watson et al., for a comprehensive review [10]). Mental health difficulties, more broadly, can also disrupt parenting, and this may be a potentially modifiable mechanism of intergenerational transmission [11]. A recent systematic review of controlled studies exploring parenting attitudes, behaviours and parent–child interactions reported that parents with eating disorders may experience higher levels of parenting stress than parents who do not have an eating disorder, and in non-feeding contexts they may, on average, be more intrusive, less sensitive and provide less facilitation when interacting with their children [12]. In addition, these parents may differ from controls in specific eating disorder-relevant domains. For example, studies suggest that parents with eating disorders may experience greater concern about their children’s weight, and parent-child mealtime interactions may be characterised by increased conflict [12].

Despite these findings, a relatively small number of studies have explored the lived experiences of parents with eating disorders. An accumulation of these studies is vital if we are to understand the complexities of parenting with an eating disorder and find effective ways to support parents affected by these serious conditions, and their children. Of the qualitative studies that have been conducted in this area, many authors report that mothers with eating disorders may experience significant challenges, from pregnancy and onwards through parenthood. Common findings described in these studies centre around conflicts of identity [13–15], and battles between the demands of an eating disorder and the needs of children [6, 16–18]. Several studies also suggest that mothers with eating disorders may doubt their parenting abilities or feel inadequate in the maternal role [14, 17–19], and be beset by feelings of guilt and shame [15, 18, 19]. Mothers with eating disorders also describe tangible concerns in relation to the potential impacts of their illness on their children, including fears specifically around intergenerational transmission [5, 6, 15, 16, 19, 20]. A range of difficulties around feeding and food-related activities in the context of family life have also been described [5, 17, 20, 21], along with maternal concerns about children’s weight [18, 21].

To date, the majority of qualitative studies in this area have focused on specific periods related to parenthood (e.g. pregnancy, or mothers of very young children); on specific phenomena such as feeding perceptions; or they have recruited participants from clinical settings. To our knowledge, only one qualitative study, conducted in Australia by Stitt & Reupert [6], has explored the experiences of parents with either a current or previous eating disorder diagnosis, who have children of any age, and who were recruited from a community sample. We sought to take a similarly broad approach to recruitment to further explore and give voice to the experiences of parents with a range of eating disorder diagnoses, including binge eating disorder, which until now has been largely neglected in the qualitative literature on parental eating disorders. Based on the extant literature, our aim was to extend the depth of our knowledge in this area by asking parents about their lived experiences in both eating disorder-specific and more general parenting domains. We also sought to identify the support needs of parents, both for themselves and for their families, in their own words, and we were interested in understanding parental perceptions of how their eating disorder impacted on their families, and their children in particular. To enable comprehensive coverage of both of these separate but closely interlinked topics, we present our findings in relation to the impacts of having an eating disorder on parents here, and we report on parental perspectives of the impacts of having an eating disorder on children and around intergenerational transmission in a subsequent paper [22].

## Method

This study employed a qualitative methodology to explore the experiences of parents with eating disorders. Semi-structured interviews were conducted online by the first author (LC), with adults reporting a broad range of eating disorder diagnoses. The research team comprised a PhD student with lived experience of  an eating disorder (LC), and two clinical and developmental psychology researchers (SC and KL), one of whom is also a clinical psychologist (SC). A reflexivity statement is provided in Additional file 1. Prior research findings [12] informed the development of the interview schedule employed in the current study. Interview questions were designed to be open-ended and an effort was made to phrase these in a non-directive way. Data were analysed using an inductive approach to reflexive thematic analysis [23], and the study is reported in line with the Journal Article Reporting Standards (JARS; [24]) for qualitative research.

### Recruitment

Individuals were eligible to participate in the study if they were over the age of 18 and were the parent to one or more children aged two years or older. A minimum age of two years for children was specified as this age group has been relatively neglected in the literature, and because infants under the age of two tend to require different parenting to older children and adolescents. Participants were required to report having received an eating disorder diagnosis from a health professional at some point in their life (although evidence of this was not required), and to have experienced eating disorder symptoms since becoming a parent.

Participants were recruited between March 2021 and November 2021 through social media advertisements, including online community groups, parenting groups, and eating disorder support groups. The lived experience of the interviewer (LC) was highlighted in participant information sheets and study advertisements. Parents responded to a brief eligibility survey, which also captured self-reported eating disorder diagnoses, and eligible participants who provided their contact details were invited to an online interview via email. Eighteen participants signed up for an interview.

Prior to interviews, parents completed an online consent form and brief demographic questionnaire. Participants were also asked to report any non-eating disorder mental health diagnoses they had received, and to indicate whether they had experienced symptoms relating to any of these diagnoses since becoming a parent. Following each interview, participants received an email thanking them for their time, which included links to relevant eating disorder and mental health support services.

The study received ethical approval from the University of Sussex Sciences & Technology Cross-Schools Research Ethics Committee (Review Number: ER/LAC25/8). Procedures were in place should any safeguarding concerns arise, and this was explained to participants at the start of each interview. Before interviews commenced, participants were asked verbally if they consented, in addition to the consent they had previously provided via the pre-interview questionnaire.

### Participants

A total of eighteen mothers aged 30–48 years (*M* = 38.5, SD = 3.94) participated in the study. The majority of participants were based within the UK (*n* = 12), with other mothers participating from the USA (*n* = 5) and Australia (*n* = 1). Across the sample, there were a total of 36 children aged between two and seventeen years (*M* = 9.67, SD = 3.92). 52.78% (*n* = 19) of children were male, while the remaining 47.22% (*n* = 17) were female.

Most of the mothers who participated relayed a long history of eating disorders, with the majority (*n* = 12; 66.67%) having first received an eating disorder diagnosis prior to having children. Just over 27% of participants (*n* = 5; 27.78%) reported that they were currently accessing treatment for their eating disorder at the time of interview. Just over three quarters of participants (*n* = 14; 77.78%) had received additional diagnoses relating to their mental health, and 71.43% (*n* = 10) of these participants had experienced symptoms associated with at least one of these additional diagnoses since becoming a parent.

Participant demographic characteristics are presented in Table 1, with self-reported diagnostic information presented in Table 2.

**Table 1:** Participant demographics

Characteristic	*n*	%
**Gender**		
Female	18	100
**Relationship status**		
Divorced	1	5.56
Single	2	11.11
Married/co-habiting/in a civil partnership	15	83.33
**Ethnicity**		
White–British	12	66.67
Another White background	6	33.33
**Employment status**		
Full-time employed	10	55.56
Self-employed/freelance, student, stay at home parent, or unemployed	7	38.89
Prefer not to say	1	5.56
**Highest educational qualification**		
Completed secondary education	5	27.78
Undergraduate degree or equivalent	7	38.89
Postgraduate degree or equivalent	6	33.33
**Number of children**		
1	3	16.67
2	13	72.22
3	1	5.56
4	1	5.56

**Table 2:** Eating disorder and other mental health diagnoses

Diagnosis	*n*	%
**Eating disorder diagnoses**		
Anorexia nervosa	5	27.78
Bulimia nervosa	4	22.22
Binge eating disorder and bulimia nervosa	2	11.11
Binge eating disorder	1	5.56
Anorexia nervosa and bulimia nervosa	1	5.56
Bulimia nervosa and EDNOS	1	5.56
Binge eating disorder and EDNOS	1	5.56
Bulimia nervosa and OSFED	1	5.56
Bulimia nervosa and ARFID	1	5.56
EDNOS	1	5.56
**Additional mental health diagnoses**	Count ^a^	
Depression	9	
Anxiety	3	
Generalised anxiety disorder	2	
Post-traumatic stress disorder	2	
Social anxiety	1	
Obsessive compulsive disorder	1	
Seasonal affective disorder	1	
Dysthymia	1	

ARFID = Avoidant/Restrictive Food Intake Disorder; EDNOS = Eating Disorder Not Otherwise Specified; OSFED = Other Specified Feeding or Eating Disorder

^a^Count for additional mental health diagnoses refers to the number of times each diagnosis was reported across the 14 participants who reported diagnoses in addition to an eating disorder

### Data collection

The interview schedule was developed by the research team and can be found in Additional file 2. Data was collected online using Zoom between April 2021 and November 2021. Interviews were audio recorded and transcribed verbatim. Interview length ranged from 35 to 97 min, with an average interview duration of 51 min.

Data collection was halted after eighteen interviews. In consideration of current debate concerning data saturation in reflexive thematic analysis (see [25]), the decision to cease data collection was informed by the information power model outlined by Malterud et al. [26]. It was felt that after eighteen interviews the sample held sufficient information power. Whilst the study had a broad aim and analysis was to be conducted across cases, the dense sample specificity and strong dialogue between the participants and the researcher, along with background theoretical knowledge of eating disorders and parental mental health difficulties, informed the decision to cease data collection. It was also felt that following eighteen interviews the participants that had been recruited represented a sufficient range of different eating disorder diagnoses.

### Data analysis

An inductive, critical realist approach to reflexive thematic analysis [23] was employed to analyse the data. Following transcription, each interview was coded by LC using NVivo 1.6.1. software [27] for any information relevant to the topic of having an eating disorder in the context of being a parent. Initial codes were descriptive and extensive, and were generated by a thorough absorption of the data which began at the point of transcription. Two rounds of coding of the full dataset were conducted. Codes were then grouped into potential themes and subthemes. The thematic structure was developed iteratively, with constant review of the corresponding data and through regular discussion between LC, SC and KL. Themes and subthemes were checked for the number of participants represented by each, to ensure these were as representative as possible of the participant group as a whole. In the context of reflexive thematic analysis, and in light of the sensitive nature of the topic, member checks were not carried out for this study (see Varpio et al. [28] for a discussion of limitations associated with member checking in reflexive qualitative research).

Following analysis, it was evident that a number of themes pertained more closely to parental experiences of having an eating disorder, whilst others were more closely related to issues concerning perceived impacts on children, and intergenerational transmission specifically. To enable a rich, in-depth exploration of both aspects, a decision was taken to present these in separate reports. Only the themes relating to parental experiences of having an eating disorder are reported here.

## Results

A total of four themes relating to parental experiences were identified, each with multiple subthemes. Overall, while parenthood could motivate eating disorder recovery, mothers experienced a range of parenting challenges in the context of their eating disorders. Participants highlighted a number of barriers to accessing support, and identified ways that services could better serve their needs. Illustrative quotes for each theme are presented below in abbreviated form,^1^ and all names are pseudonyms created using a random name generator. Additional quotations, along with the number of participants represented within each subtheme, can be found in Table 3 (Additional file 3).

### Theme 1: Parenthood as a double-edged sword

This theme captures the ways in which mothers described the parental role as impacting on eating disorder symptomatology, both negatively and positively. On one hand, participants talked about unique and diverse eating disorder triggers they encountered in the context of being a parent. On the other hand, whilst at different stages of recovery, mothers often spoke of the ways that parenthood had inspired and continued to motivate them to recover from their eating disorder.

#### 1a. Parenthood presents unique eating disorder triggers

For some of the mothers, changes associated with becoming a parent had contributed to the development or worsening of eating disorder symptoms. These included changes to the body occurring through pregnancy, as well as changes more closely related to identity. Some of these mothers also commented on the impact of conversations and comments around weight and shape in relation to their post-pregnancy bodies.

But… there was a compounding effect of being a single parent, and the psychological and emotional issues that went with that, that I think fed into the causes of the eating disorder as well… there's issues around how your body looks, but there's also issues around not being who you were… (Georgina)

In addition to the influence of becoming a parent on eating disorders, participants described a wide range of external triggers that they encountered specifically in the context of their parental role. Children themselves could trigger eating disorder behaviours, either unknowingly via their own bodies or inadvertently through their words. Family conflicts, lack of sleep, and activities with or by the children that involved food, such as cooking brought home from school, children’s birthday parties, and going out for ice-cream in the summer, were also noted as parenting-related triggers for eating disorders.

I've struggled as my daughter has gotten older. She's very, very, very slender so it's like an added trigger that's baked into the family, where you struggle with that. You feel guilty, like oh my god, she's my child, but I'm looking at her and she's this little itty bitty thing… (Isabel)

A number of participants reported that the day-to-day stresses associated with parenting, beyond specific triggers related to becoming and being a parent, could also impact eating disorders.

I think a lot of times, they're [eating disorder services] dealing with 20-year-olds that don't necessarily have kids, and so, [being a parent] just kind of would get ignored. That's an extra stressor and tension in day-to-day life that isn't even talked about… How to deal with those things, yet continue to want to take care of yourself, I don't know. (Gabrielle)

Many mothers described challenges relating to planning family meals and eating together as a family, and some spoke of engaging in eating disorder behaviours to compensate specifically for actions designed to reduce the perceived risk of children picking up eating disorder-related attitudes and behaviours.

… part of the reason why I purge is because I am indulging in non-healthy choices, in order to make sure that I don't pass on something to [child]. (Freya)

#### 1b. Children motivate recovery

Although the parental role presented a range of specific challenges, several participants also reported that being a parent was integral to their recovery journey. Children had both prompted the process of recovery, and provided ongoing motivation. In some cases, this motivation stemmed from mothers’ desires to improve their children’s experience, which they perceived to be lacking as a result of growing up with a parent who has an eating disorder.

I think one of my main motivators to work towards recovery and to be in recovery, is my children, and just seeing that in the midst of my disorder, the life that I was - am - providing for them is not how I ever pictured their life being or the kind of mum I would want to be. (Gabrielle)

In other cases, this motivation sparked from participants’ conscious concerns that their children might otherwise develop eating disorders themselves.

And it all started when I was a child. And I have a son and a few years ago I saw some of my patterns being mirrored in him. And that was the moment that I finally decided to get therapy for my eating disorder. (Hannah)

### Theme 2: The eating disorder impacts on parenting

Mothers reported that having an eating disorder impacted on parenting in many different ways. Mothers spoke about ways that, at times, their eating disorder could assume control. Having an eating disorder was often described as amplifying the typical challenges associated with parenting. Parents also spoke of the more specific ways that having an eating disorder could impact parenting, including descriptions of being less physically present, a sense of being less present psychologically, and experiencing negative moods which could sometimes affect interactions with children. Mothers also spoke of a range of ways that having an eating disorder specifically impacted food-related parenting.

#### 2a. The eating disorder in control

Participants spoke candidly about how, at times, their parenting could differ during periods of illness compared to periods of remission. Some mothers reported occasions where the eating disorder, or the compulsions associated with it, would take over and take precedence over momentary parenting decisions.

… I wasn't always able to put their needs first. Not because I was distracted from what their needs were necessarily but because… the compulsion to do what I had to do, had to come first, so I wasn't prioritizing my children in the moment. And that wasn't all the time. But when I had to do whatever I do, then that's what I had to do, and so I wasn't always putting their needs first. (Esther)

Relatedly, participants spoke about the ways in which parenting expectations could, at times, aggravate and interfere with the demands of the eating disorder, and of needing to schedule eating disorder behaviours around childcare responsibilities so as to meet these conflicting demands. This was noted most commonly in relation to eating disorders where bingeing and/or purging was a feature.

… not every night, but at bedtime… if she doesn't wanna go to sleep, it's like I can feel myself getting irate. Because if my partner's out, and she doesn't wanna go to sleep and I'm thinking… this is my time to eat girl, go to bed… I find myself getting sort of irate with her, losing my temper a little bit more… 'cause I'm just frustrated. (Carina)

#### 2b. The eating disorder magnifies the challenges of parenting

Similarly to the ways that parenting could exacerbate eating disorder symptoms, many parents reflected on the ways that having an eating disorder could amplify the challenges associated with parenting.

All these things that people don't know go on in a day of these mental gymnastics of can I allow this, can I have that, is that safe, is that not safe… it's very exhausting... Being a parent you already have a level of anxiety that you're responsible for people… it's all mixed in, they look to you for everything so that, I think the level of feeling like the world's on your shoulders is heightened a little bit more. (Isabel)

#### 2c. Reduced capacity for activities with the children

Participants frequently reported that their ability to participate in a range of activities with their children was significantly reduced during periods of illness. Often this was as a result of the physical effects of eating disorders, such as an inability to regulate one’s temperature when outside, or fatigue related to dietary restriction.

Things like swimming, being outside. ‘Cause I couldn't cope with the cold. I'd lost so much weight so fast. I was so cold, and the boys always wanted to go to the park after school, and I had to say "I'm really sorry boys, I can't manage today". (Bethany)

Some parents reported non-physical reasons related to their eating disorder for their reduced capacity for activities with their children, such as negative body image, or not being able to participate in food-related activities. Sometimes, this reduced capacity to engage with children resulted from negative moods, leading to a tendency to withdraw and self-isolate.

I also tend to kind of withdraw and not care. So I spent a lot, most of the waking hours… I would feed them, and then kinda come into this room and just sit here by myself and not want them around. (Gabrielle)

In addition, a few of the mothers reported changes in the way the family operates when they are unwell compared to when they are more well. This included both a change to the general nature of the family’s experience, as well as more defined changes in family roles, such as children taking on caring roles, or partners taking on new responsibilities while the mother was unwell or away from the family for treatment.

… it was almost that tunnel vision and the children were there and I could just about, just about, do enough to [EXHALES]... make sure they were safe basically… And anything beyond that was just a luxury that I couldn't engage with, so that became my husband's role. (Bethany)

#### 2d. Not being ‘present’

In addition to not being physically present at times, many participants reported experiencing their eating disorder as all-consuming, taking away mental space and energy they felt might otherwise be available for their children. Mothers reported a sense of not always being ‘present’ for their children, and conveyed feeling that their children may have missed out as a result.

I remember saying one time to a therapist that, even if I was with her 24/7, I'm not sure I was ***with*** her... do you know what I mean? … I was there. Maybe thinking about something else, maybe eating, running to the toilet. Of course I was there, but I was not... bonding with her. (Catherine)

#### 2e. Experiencing fractious interactions with children

Many parents reflected on the ways in which eating disorder symptoms affected mood states, which on occasion could impact subsequent interactions with children. Participants spoke of times where they might feel stressed, frustrated, or annoyed, and of how they might find themselves snapping or losing patience with their children. Often, participants related these emotions to symptoms associated with their eating disorder, such as dietary restriction, or negative body image.

I'll find myself snap. I'm like "What are you doing? Don't do that" [to child]… and then I think, oh god why have I just said that to her? But it's 'cause I am feeling so sort of wound up and anxious because I had to go and put clothes on. (Carina)

#### 2f. Impacts on food-related parenting

Participants reported that having an eating disorder impacted on food-related parenting in a range of different ways. Many mothers reported avoiding situations involving eating as a family, and some described measures taken to manage their eating disorder which would subsequently influence the foods the rest of the family ate. These included measures put in place to adhere to eating disorder ‘rules’, or measures learnt over the course of recovery. A small number of participants reported disagreements with partners over their children’s eating, and some mothers spoke of a particular consciousness about their children’s diets, which was on occasion, after reflection, viewed as a positive aspect of being a parent with an eating disorder.

I'm always conscious… whatever I'm putting on the table... is within my limits. If I know something's got fifty grams of fat in for example, like a takeaway or something like that, then it's not gonna happen. And that's as much for me as it is for the children, because I'm conscious of what they put in their mouth as well... (Charlotte)

### Theme 3: Blame and burden

Theme 3 captures the emotional experiences of being a parent with an eating disorder. Mothers talked about experiences of being judged negatively; both by others and by themselves. They spoke about experiences of guilt and shame, as well as sadness and regret.

#### 3a. Judgement

A number of participants described being judged negatively and treated without compassion, either by healthcare professionals or by family members for having an eating disorder whilst also being a parent.

The hardest thing, when I was in hospital, was being made to feel small and unworthy and… you know, "What on earth are you doing? You're a parent, you've got children, how could you do this to them?". (Charlotte)

Several mothers spoke about feeling that they were in some way to blame for their illness, and there was a sense of self-stigma: a perception that by being older and with children, they weren’t ‘typical’ eating disorder sufferers.

I'm not... a teenager anymore where it's socially acceptable to have this problem. And so you get into all those societal issues where it's like, you feel the embarrassment is so high of “yeah I struggle with this, this is a problem”. That was kind of the biggest thing for me, just coming out with that. (Isabel)

#### 3b. Guilt and shame

Relatedly, many mothers spoke of experiences of guilt and shame surrounding their eating disorder in the context of being a parent. Feelings of guilt were reported in relation to specific events, such as guilt over time taken away from the family for treatment, or guilt about taking an infant to a therapy session in the absence of alternative childcare. Other experiences of guilt were more holistic, and were related to the very fact of having an eating disorder as a parent, or to the effect that this had on parenting. Parents expressed shame over parenting, on occasion, in ways when unwell that they would not choose to parent during periods of remission.

There's so much guilt involved with eating disorders as well… huge amount of guilt that, if I was eating better, if I didn't have this, I was more in control, that I’d have more energy, I'd be able to do more with the kids. (Nadia)

#### 3c. Sadness and regret

A few of the participants explicitly expressed sadness and regret around having an eating disorder, in relation to the perceived impacts of this on their children and family life.

I think that it had begun with… breastfeeding both of my kids and, it's something that I did do, but I think that… I wasn't able to do it, in either case, as long as I would have liked since I was too eager to lose the pregnancy weight… which I did and I guess at the time I was… relieved about but, I… do still kind of regret that actually. (Andrea)

### Theme 4: Seeking support

This theme captures the experiences of participants as they talked about seeking support for their eating disorder as a parent, and their hopes for future support opportunities. Mothers talked about a lack of awareness and information around parenting with an eating disorder, and described a range of barriers to accessing treatment that were unique to being a parent. Participants reflected on what they wanted to gain through support, and made suggestions as to how some of these goals could be operationalised.

#### 4a. Lack of awareness and information

Mothers frequently reported that there was little awareness about or guidance for parents with eating disorders. This included both guidance for parents and families themselves, and awareness and guidance for professionals working with adults experiencing eating disorders, whom participants perceived were more used to supporting adolescents.

It's [being a parent] a topic that never really came up, as I was going through my eating disorder. It was treated as though it was irrelevant, and I don't think it is irrelevant, at all. (Georgina)

#### 4b. Unique barriers to treatment

Many participants spoke about eating disorder treatments as being designed with adolescents in mind, and of difficulties accessing treatment as a parent. These difficulties were evident both in terms of finding ways to balance treatment alongside parenting and other responsibilities, and in relation to a lack of dispensation for parents requiring inpatient treatment when treatment *was* accessed.

Particularly in the last maybe two years or so, in trying to seek treatment as a parent there's not much that I've been able to find that I'm able to co-ordinate with having a career and children… (Andrea)

Several participants reported fears that if they sought treatment as a parent with an eating disorder, there would be a risk that their ability to look after their children would be called into question by professionals in positions of authority. This seemed to be a very real potential barrier to parents accessing support.

When I mention it to anybody, you know about the depression or about the eating disorder, it does worry me that people will think... "She’s not fit to be a parent. It's quite worrying, the things that she's saying", and then all of a sudden there's people knocking on my door saying "You can't be a mother"... That's my biggest concern. (Victoria)

#### 4c. Support goals

Participants talked about how they wanted tailored support for parents with eating disorders. This included greater support for managing their eating disorder, which mothers felt might in turn benefit their families and children. Parents highlighted the importance of support provided by professionals being non-judgemental.

I think it would just be… an understanding, and a feeling of... acceptance that it's okay to have an eating disorder and be a parent. It's, you know, “you're not gonna be judged for it, we're gonna help you, we'll support you. And we'll support you just like we would support a teenager.” (Charlotte)

Many of the mothers spoke about desiring connection with other parents who have eating disorders. A number of reasons for this desire were highlighted, including to normalise the experience, to reduce a deep sense of isolation, and to offer hope.

I think [EXHALES]... the biggest thing would be that sense that you weren't utterly alone and utterly abnormal in your experience. That I wasn't the only parent, with kids, who has an eating disorder. I'm not the only parent with kids that chooses to talk to them about it or, if somebody chooses not to, that they're not alone in that either. I guess it's just that sense of representation and not being an absolute freak of nature and failure as a parent as well. (Bethany)

#### 4d. Practical support suggestions

Participants spoke about the need for specialised support. Support suggestions included guidance for parents, such as strategies for coping when the eating disorder is triggered by parenting, as well as guidance on how to interact with children around eating disorder-relevant areas. Mothers spoke frequently about the potential value of professionally facilitated peer support groups.

I think support from other parents and knowing that you're not alone is always very helpful. That kind of peer support, and you're not weird and you can get better and… eating disorders I guess can be really lonely, and actually having support from other parents who've been through it is so important in parenting full stop. So actually, in the context of an eating disorder I could see how that could be really powerful. (Esther)

Several participants additionally expressed a desire for support for the wider family, including support in the form of family counselling or facilitated family support groups. Some mothers also spoke about the potential for support groups specifically for children which might help them to understand their parent’s illness and could also provide an avenue for their own mental health and wellbeing to be supported. Some parents, on the other hand, felt that it wouldn’t be appropriate for their children to be involved in an intervention, and others indicated that children’s involvement should be dependent on the children’s age.

Help for the whole family, 'cause obviously the eating disorder doesn't just affect the one who's got it. It affects the whole family. It affects the other half. It affects the kids. It affects ***my*** parents… It affects the whole family, so I wish there was a way that the whole family could get support. (Lina)

## Discussion

This study explored in-depth the lived experiences and support needs of mothers previously or currently diagnosed with an eating disorder who had experienced symptoms since becoming a parent. To broaden and extend the existing knowledge base in this area, we recruited an international sample of parents with a range of eating disorder diagnoses, who had children of any age over two years.

The themes described within the present report, which pertain specifically to impacts on the parent, suggest a complex and bidirectional relationship between parenting and eating disorders. For our participants, parenthood not only impacted eating disorders, but eating disorders impacted parenting, in a range of different ways. These impacts were both specific (such as reduced capacity to engage in activities with children) and more general (for example, the stresses associated with being a parent were heightened in the context of having an eating disorder). In addition, the mothers in our study spoke of experiences of judgement, both externally and internally, and the experience of being a mother with an eating disorder was frequently accompanied by a strong sense of guilt, shame, and sadness.

Our comprehensive approach offers new insights into the particular challenges adults with eating disorders describe in the context of being a parent. A wide range of eating disorder triggers that are specifically related to being a parent were identified, beyond those relating to food and eating. Children’s bodies and words, family conflicts, attempts to hide eating disorder behaviours from children, and the day-to-day stresses of parenting presented unique triggers for the mothers who participated in this study. Simultaneously, these day-to-day stresses were amplified in the context of having an eating disorder; a finding which is in line with the results of quantitative studies suggesting that mothers with eating disorders may experience higher levels of parenting stress compared to control mothers [29–31].

Our analysis also highlights the myriad of ways that having an eating disorder can impact on parenting activities, and casts light on the ways that these impacts can extend beyond food and eating-related contexts. Specifically, mothers spoke of the changes that occur within the family unit during periods of illness, of being unable to participate in a range of different activities with their children, and of how some of their interactions with their children could be impacted. To quote one of our participants, “it’s not about the food”. Where possible, future studies investigating the effects of parental mental health difficulties on children should consider a broad spectrum of potential impacts that extend beyond the most salient behavioural features of those difficulties – in this case, beyond attitudes and behaviours around food, eating, and the body.

In addition, a number of our findings echo those described by other qualitative studies in this area. For example, the finding that - despite presenting additional challenges - parenthood provided motivation for many of the mothers in our sample to pursue recovery from their eating disorder has been reported elsewhere [6, 15, 21]. This corroborated finding has important implications, as the motivating force of motherhood may present a specific opportunity for women with eating disorders to seek treatment and support, at a time in their life when they are more likely to be in regular contact with health services. The readiness of such services to respond sensitively and without judgement will likely determine the extent to which this opportunity can be utilised. With regards to the provision of services for parents with eating disorders which break down the barriers to engagement highlighted within our study, there is still a lot of work to be done.

Conflicts between the demands of an eating disorder and the needs of children have also been documented in several previous studies [6, 16–18]. A sense of sometimes not being psychologically present as a result of eating disorder-related preoccupation was frequently mentioned by the mothers in our study, and has also been identified previously [6]. Various impacts on food-related parenting have also been described in existing qualitative studies [5, 17, 21], as have the powerful experiences of guilt and shame described by this particular group [15, 18, 19]. Although replication was not an a priori objective of our study per se, these converging findings, which have been identified across a range of different contexts and utilising a range of different qualitative analytic strategies, support the extant literature indicating that mothers with eating disorders can experience numerous challenges in the context of parenthood (see [32] for a discussion of replication in qualitative research).

## Limitations

Whilst giving voice to a seldom-heard group, our study was not without limitations. Although our participants spanned three continents, our sample was comprised exclusively of White mothers from Western countries, and thus does not represent the experiences of parents from other ethnic backgrounds, nor other countries. Neither does it capture the experiences of fathers with eating disorders. Although the present study was open to parents of any gender, only mothers registered their interest in participating. Many men with eating disorders will be fathers, and there is a need to find innovative ways to engage fathers in both parenting and eating disorders research.

It is important to note that comorbidity with other mental health diagnoses was high within our participant sample. Such comorbidity is commonly observed in eating disorders [33], and whilst we consider it a strength of our analysis that only information that was considered relevant to both being a parent and having an eating disorder was coded, it is feasible that some items may have been more closely related to comorbid symptoms, rather than eating disorder symptomatology. For example, several mothers described their reduced capacity for activities with their children during periods of illness. Whilst in many cases this was attributed directly to having an eating disorder by participants, sometimes it was described in relation to a tendency to withdraw and self-isolate. This withdrawal may well have been associated with having an eating disorder, but equally could result from symptoms more closely aligned with depression, for example. Future research may wish to carefully pick apart the ways that specific mental health symptoms might affect parenting.

### Clinical implications

For the mothers who participated in our study, parenthood is fraught with triggers and challenges that standard treatments for eating disorders currently fail to consider. These findings highlight the importance of practitioners knowing about and understanding the family contexts within which individuals live and how these factors can influence symptomatology. However, when asked, only two out of the eighteen mothers who participated in our study reported having ever been asked by a health professional about being a parent in the context of their eating disorder.

There is a need for accessible and specialised treatment options for parents, which acknowledge the myriad of challenges associated with parenting with an eating disorder and that provide support for this in a non-judgemental way. Whilst our study explored the experiences of mothers with children aged two years or older, our findings are not dissimilar to some of those identified in qualitative studies of women with eating disorders at earlier stages of parenthood (see [34] for a recent review). Support for parents with eating disorders should be offered from an early stage, and continue to be offered as these mothers navigate the challenges of different stages of parenthood, from pregnancy onwards.

As children get older, family-based approaches may in some circumstances help to alleviate some of the concerns parents have about the effects of their eating disorder on the family unit, and peer support options may be one way to reduce the isolation many of the mothers who participated in this study described, whilst simultaneously reducing the stigma associated not only with having an eating disorder, but being an adult - and a parent - with an eating disorder. Accessible guidance and information around managing parenthood whilst also managing an eating disorder may be one way to empower parents with eating disorders and potentially reduce harmful self-judgements leading to feelings of guilt and shame, which may ultimately perpetuate eating disorder symptomatology [19].

## Conclusions

The relationship between eating disorders and parenting, as relayed through the stories of mothers with a range of lifetime diagnoses, is multi-faceted. While parenthood may motivate recovery, it can also present a range of unique challenges, and having an eating disorder can impact parenting in ways that extend beyond the domains of food, eating and the body. Mothers with eating disorders may additionally experience guilt, shame, and sadness, and feel that their needs are not met by standard eating disorder treatment services. Specialised, non-judgemental support for parents with eating disorders and their families, and access to peer support opportunities may help to reduce stigma and isolation, and ultimately help parents to confidently manage the challenges of parenting with an eating disorder.

### Supplementary Information

**Additional file 1**. Reflexivity Statement. Statement of reflexivity from the authors.

**Additional file 2**. Interview Schedule. Copy of the Interview Schedule used for interviews.

**Additional file 3**. Themes and subthemes with example quotations. Table summarising the themes and subthemes identified during analysis, along with examples from the data and the number of participants represented within each subtheme.

## Data Availability

Due to the constraints of the ethical approval gained for this study, interview transcripts are not publicly available.

## Abbreviations

UK: United Kingdom
USA: United States of America
LC: Author 1
SC: Author 2
KL: Author 3
JARS: Journal Article Reporting Standards
ARFID: Avoidant/Restrictive Food Intake Disorder
EDNOS: Eating Disorder Not Otherwise Specified
OSFED: Other Specified Feeding or Eating Disorder

## Footnotes

1 Potentially identifying information has been removed, and non-word utterances have been deleted, where applicable. Grammar has also been adjusted where necessary to improve readability, but the context and content of quotations has not been altered in any way.
