# Supplementary material for: “I think it is woven through me, and sadly that means it is woven through our family life”: the experiences and support needs of mothers with eating disorders
Source: J Eat Disord. 2023 Aug 29;11:147. doi: 10.1186/s40337-023-00868-y (PMC10466810; doi:10.1186/s40337-023-00868-y)
Supplement: Supplementary file 1 — Additional file 1. Reflexivity Statement. Statement of reflexivity from the authors. [file 40337_2023_868_MOESM1_ESM.docx]

**Reflexivity Statement**

The authors acknowledge the ways in which the identities and personal experiences of the research team can impact data collection, analysis and interpretation in qualitative research. A PhD student (LC) conducted all data collection, and led the analysis and interpretation of the data. LC has a long-standing research interest in eating disorders. She also has personal experience of an eating disorder and this lived experience was highlighted to potential participants in recruitment materials and participant information sheets. LC reflected that during the process of data collection, she felt this lived experience facilitated a strong rapport within interviews, perhaps through the provision of a supportive environment in which participants could share their experiences freely. LC approached interviews and data analysis with understanding and empathy, but she is not a parent herself, nor does she identify as being the child of a parent with an eating disorder. LC consulted with SC and KL regularly throughout the process of data collection, analysis and interpretation, openly sharing her reflections on the data and how, at times, certain aspects of the data collected was relatable in terms of her own experience of an eating disorder.

SC is an academic clinical psychologist with a research interest in the intergenerational transmission of mental health. She is the parent of a teenage daughter. During the analytical process and during the discussing of themes emerging from the interviews, SC kept in mind that her own experiences as a parent and academic may have influenced her response to the data.

KL is a developmental psychologist with a research interest in parenting and the intergenerational transmission of mental health disorders. She is a parent of two school-aged children, one of whom has disordered eating as a consequence of OCD. During the analytical process and discussions regarding emerging themes with LC and SC, KL maintained an awareness that her own experiences as a parent and a researcher may have influenced how she interacted with and interpreted the data.
