# Supplementary material for: “I think it is woven through me, and sadly that means it is woven through our family life”: the experiences and support needs of mothers with eating disorders
Source: J Eat Disord. 2023 Aug 29;11:147. doi: 10.1186/s40337-023-00868-y (PMC10466810; doi:10.1186/s40337-023-00868-y)
Supplement: Supplementary file 2 — Additional file 2. Interview Schedule. Copy of the Interview Schedule used for interviews. [file 40337_2023_868_MOESM2_ESM.docx]

**Interview Schedule**

1. **Can I ask you first to tell me why you were interested in taking part in this study?**
2. **Would you also be able to tell me a little bit about your family situation, in terms of how many children you have, how old they are, and whether you are a single parent or not?**

Follow-up questions:

When the family includes older children: Do the older children still live at home?

When the relationship situation is unclear: Do you live in separate homes?

1. **Would you also be able to talk about your eating disorder diagnosis, for example, when you were first diagnosed and what you were diagnosed with?**

Follow-up questions:

Have you ever accessed treatment for your eating disorder?

Are you currently accessing treatment?

1. **Have you ever been asked about being a parent in the context of your eating disorder, for example by a health professional when you were diagnosed/during treatment?**

Follow-up questions:

What did you think about being asked about that?

Did it lead to any additional support or advice?

The next few questions are about whether your eating disorder impacts on your parenting and family life in any way. Some people feel that their eating disorder does impact on parenting but this isn’t the case for everybody, so there are no ‘right’ or ‘wrong’ answers.

1. **Thinking about your own experience of being a parent, do you feel that there are any aspects of parenting that are particularly challenging when you have an eating disorder?**

Prompt (if needed): What about situations you encounter as a parent that might be related to food, or eating, or perhaps body image?

IF challenges identified:

Can you tell me a little bit about any times when it’s felt as if these difficulties might impact on your children/family?

IF yes:

What are your thoughts about whether these difficulties impact your son(s) and daughter(s) in similar ways?

How, if at all, do you feel that these difficulties are different when it comes to your older compared to your younger children/Do you feel that these difficulties have changed as your child has got older?

Follow-up question:

What impact, if any, has the Covid-19 pandemic had on these aspects of parenting?

1. **How might having an eating disorder impact on your parenting more generally, if at all?**

Prompt (if needed): For example, some people find that having a mental health difficulty can make them a little preoccupied or more stressed or distracted, and this could perhaps make it more difficult to be firm about household rules, for example.

IF challenges identified:

Can you tell me a little bit about any times when it’s felt as if these difficulties might impact on your children/family?

IF yes:

What are your thoughts about whether these difficulties impact your son(s) and daughter(s) in similar ways?

How, if at all, do you feel that these difficulties are different when it comes to your older compared to your younger children/Do you feel that these difficulties have changed as your child has got older?

Follow-up question:

What impact, if any, has the Covid-19 pandemic had on these more general aspects of parenting?

1. **How might having an eating disorder have had an impact on your relationship with your**

**partner and/or wider family, if at all?**

IF negative impacts identified:

Have there been times when it’s felt as if these difficulties might impact on your children/family?

Follow-up question:

What impact, if any, has the Covid-19 pandemic had on these relationships?

1. **What strategies, if any have you found helpful for dealing with any challenging feelings,**

**thoughts or situations you’ve encountered as a parent with an eating disorder? These could**

**be parenting strategies, or coping strategies you’ve used for managing your eating disorder**

**in the context of being a parent.**

IF strategies identified:

Can you talk a bit more about why you found that helpful?

IF no strategies identified:

Is there anything you’ve tried that *hasn’t* worked?

Why do you think that didn’t work?

1. **If an intervention could be developed to support parents with eating disorders, what do you think are the most important things it should include?**

Follow-up questions:

Would you value general parenting support and advice? For example, advice around

managing children’s difficult behaviour?

Would you value support and advice specifically in relation to contexts that might be particularly challenging for parents with eating disorders? For example, advice around managing mealtimes?

What do you think would be the best format for this kind of intervention? Do you think it should be delivered online or in-person? [If in-person] do you think the support should be provided individually or within a group? Do you think you’d want to attend something just once, or access support over multiple sessions?

1. **What would you hope to gain from an intervention designed to support parents with eating disorders?**
2. **How do you think your children might benefit from an intervention like this?**
3. **What concerns, if any, would you have about an intervention developed to support parents with eating disorders?**

Follow-up questions:

What things might stop you wanting to, or being able to, access an intervention like this?

1. **Is there anything else you would like to say about being a parent with an eating disorder that we haven’t already talked about?**
