# Supplementary material for: “I think it is woven through me, and sadly that means it is woven through our family life”: the experiences and support needs of mothers with eating disorders
Source: J Eat Disord. 2023 Aug 29;11:147. doi: 10.1186/s40337-023-00868-y (PMC10466810; doi:10.1186/s40337-023-00868-y)
Supplement: Supplementary file 3 — Additional file 3. Themes and subthemes with example quotations. Table summarising the themes and subthemes identified during analysis, along with examples from the data and the number of participants represented within each subtheme. [file 40337_2023_868_MOESM3_ESM.docx]

**Table 3**

*Themes and subthemes with example quotations*

| Subtheme | Example quotations | Participants (*n*) represented within sub-theme |
| --- | --- | --- |
| **Theme 1: Parenthood as a double-edged sword** | | |
| 1a: Parenthood presents unique eating disorder triggers | - But… there was a compounding effect of being a single parent, and the psychological and emotional issues that went with that, that I think fed into the causes of the eating disorder as well… there's issues around how your body looks, but there's also issues around not being who you were… - I've struggled as my daughter has gotten older. She's very, very, very slender so it's like an added trigger that's baked into the family, where you struggle with that. You feel guilty, like oh my god, she's my child, but I'm looking at her and she's this little itty bitty thing… - I think a lot of times, they're [eating disorder services] dealing with 20-year-olds that don't necessarily have kids, and so, [being a parent] just kind of would get ignored. That's an extra stressor and tension in day-to-day life that isn't even talked about… How to deal with those things, yet continue to want to take care of yourself, I don't know. - … part of the reason why I purge is because I am indulging in non-healthy choices, in order to make sure that I don't pass on something to [child]. - I think the lack of sleep... impacts massively because that's when I'll binge... - So… last night is a perfect example… I was on track with my meal plan, I knew I needed to have an evening snack... things blew up between my [children], and it was like that level... of... just... frustration I felt inside, like put my stomach into a knot and… there was no possibility of making myself eat that evening snack... | 15 |
| 1b. Children motivate recovery | - I think one of my main motivators to work towards recovery and to be in recovery, is my children, and just seeing that in the midst of my disorder, the life that I was - am - providing for them is not how I ever pictured their life being or the kind of mum I would want to be. - And it all started when I was a child. And I have a son and a few years ago I saw some of my patterns being mirrored in him. And that was the moment that I finally decided to get therapy for my eating disorder. - But, actually it was being a parent, that drove me to, like seek help, as well. If I didn't have that motivation… I wouldn't really have had a reason to stop. Or to want to get better. - So, I tried to put up, kind of things around the house to help me stay on track with recovery and some of those involve the kids and pictures of the kids, and motivations to… stay in recovery and so they can be part of... like a motivation, I guess. A reason to stay on track. | 7 |
| **Theme 2: The eating disorder impacts on parenting** | | |
| 2a. The eating disorder in control | - … I wasn't always able to put their needs first. Not because I was distracted from what their needs were necessarily but because… the compulsion to do what I had to do, had to come first, so I wasn't prioritizing my children in the moment. And that wasn't all the time. But when I had to do whatever I do, then that's what I had to do, and so I wasn't always putting their needs first. - … not every night, but at bedtime… if she doesn't wanna go to sleep, it's like I can feel myself getting irate. Because if my partner's out, and she doesn't wanna go to sleep and I'm thinking… this is my time to eat girl, go to bed… I find myself getting sort of irate with her, losing my temper a little bit more… 'cause I'm just frustrated. - … when I read [child] her bedtime story, the thought of sitting and reading the bedtime story feels like torture. So I feel like I always gotta stand, or, move about when I’m reading it and she's like “Mum can you stand still? I'm trying to look at the pictures”…. I feel like a failure. Though I do stand still so she could see the pictures, but my head is whirring, ‘right you've gotta find another chance to exercise after that’. - …I have noticed in the past where I've gotten really agitated or really on edge where that's what I want - to engage in those behaviours - and it feels like it's being threatened and so I get very agitated and I almost do anything to get that time to myself. | 8 |
| 2b. The eating disorder magnifies the challenges of parenting | - All these things that people don't know go on in a day of these mental gymnastics of can I allow this, can I have that, is that safe, is that not safe… it's very exhausting... Being a parent you already have a level of anxiety that you're responsible for people… it's all mixed in, they look to you for everything so that, I think the level of feeling like the world's on your shoulders is heightened a little bit more. - …and maybe it's part of the disorder, I don't know, it just sometimes I guess it feels like some of the difficulties in parenting, are magnified. - …parenting's not that bad. It's really not. But then it's when there's things that aren't quite right it becomes really difficult because of the lack of support. | 7 |
| 2c. Reduced capacity for activities with the children | - Things like swimming, being outside. ‘Cause I couldn't cope with the cold. I'd lost so much weight so fast. I was so cold, and the boys always wanted to go to the park after school, and I had to say "I'm really sorry boys, I can't manage today". - I also tend to kind of withdraw and not care. So I spent a lot, most of the waking hours… I would feed them, and then kinda come into this room and just sit here by myself and not want them around. - … it was almost that tunnel vision and the children were there and I could just about, just about, do enough to [EXHALES]... make sure they were safe basically… And anything beyond that was just a luxury that I couldn't engage with, so that became my husband's role. - …from a physical health point of view, especially at my worst… I've got no energy, you feel like you're gonna pass out all the time… so I wasn't able to do things with the children. - Well [EXHALES]... a lot of the playdates are kind of hard… I need to be mindful of how much I can take it-, because it takes a lot of social energy… on my side more than the kids and, so it's... I guess it's something to do in moderation. - ... I just wanna be on my own all the time. I find it very, very difficult to… I can do things *for* the children, but to do things *with* the children is very, very hard. Even sitting, watching a film, because I just feel like I will need to retreat into my shell and, hide under the duvet and just, be by myself. | 11 |
| 2d. Not being ‘present’ | - I remember saying one time to a therapist that, even if I was with her 24/7, I'm not sure I was *with* her... do you know what I mean? … I was there. Maybe thinking about something else, maybe eating, running to the toilet. Of course I was there, but I was not... bonding with her. - I think it has been, a source of distraction, where maybe I haven't put as much energy into them and been as involved with them because I was preoccupied with, what was I gonna eat, how was I, you know… | 12 |
| 2e. Experiencing fractious interactions with children | - I'll find myself snap. I'm like "What are you doing? Don't do that" [to child]… and then I think, oh god why have I just said that to her? But it's 'cause I am feeling so sort of wound up and anxious because I had to go and put clothes on. - I'm much more irritable, I have very little patience when I'm… not eating, not nourished. - …what I live with now, rather than, the actual physical, eating disorder itself, I am left with… a negative body image about myself and it's every day. How I think I look in the mirror dictates... my mood. And if I'm having a bad time I *definitely* take it out on my children. And… my character's different… I might snap a bit more at them or a bit quicker. - So I'm not really able, I don't feel like I'm really able to deal with, like the normal aspects of my kids, like, bickering or fighting with each other really… There's no patience, I'm just... short. - …when I'm engaged in... restricting... I definitely notice… I call it, being short-fused. I'm really quick to be very explosive. | 11 |
| 2f. Impacts on food-related parenting | - I'm always conscious… whatever I'm putting on the table... is within my limits. If I know something's got fifty grams of fat in for example, like a takeaway or something like that, then it's not gonna happen. And that's as much for me as it is for the children, because I'm conscious of what they put in their mouth as well... - …when I’m being good, as it were, we all eat healthily. But when I fall off the wagon, so when I was dieting all the time, everyone would be impacted by that because it would mean “Oh let's get a takeaway tonight, screw it”. So everyone'll be eating that rubbish. - … the meal plan I'm following now… I use as guidance with them too. Everybody, when I serve dinner, everybody's getting the same portions. - We don't have food in the house... - But the… rest of the family they'd quite happily [be] eating their tea in front of a screen, but me well I'll eat… at the kitchen table, 'cause I feel safe eating on my own, sort of thing. - So… if I am restricting what I eat… it might be that we eat different food or, sometimes I just have to leave the kitchen because if I’m in the kitchen I will eat… so from a binge perspective, I can sneakily eat, quite sneakily, and like I have to remove myself from that situation which obviously means removing myself from the mealtimes. | 12 |
| **Theme 3: Blame and burden** | | |
| 3a. Judgement | - The hardest thing, when I was in hospital, was being made to feel small and unworthy and… you know, "What an earth are you doing? You're a parent, you've got children, how could you do this to them?". - I'm not... a teenager anymore where it's socially acceptable to have this problem. And so you get into all those societal issues where it's like, you feel the embarrassment is so high of “yeah I struggle with this, this is a problem”. That was kind of the biggest thing for me, just coming out with that. - …and even from the eating disorders team that I was under, you felt, I felt, judged… very much so... And that just obviously adds to your feeling of worthlessness anyway. - I always felt like I was quite old to have an eating disorder and everyone else that [eating disorder services] are seeing must be like really young… | 5 |
| 3b. Guilt and shame | - There's so much guilt involved with eating disorders as well… huge amount of guilt that, if I was eating better, if I didn't have this, I was more in control, that I’d have more energy, I'd be able to do more with the kids. - …but there is an element of guilt that, being a single parent if something were to happen to me like if I collapsed or whatever... you know, they'd be the ones to find me so it's always, you always have that guilt of feeling bad about that. - …and the blame that was put on me, for doing so [accessing inpatient treatment], was massive and something that I can never... I can't go back, but obviously it's something that I feel extremely awful about. - I suppose I have a lot of guilt from around that time, that I wasn't as present with her as I wish that I had been. | 10 |
| 3c. Sadness and regret | - I think it [the eating disorder] is woven through me, and sadly that means it is woven through our family life… - I think that it had begun with… breastfeeding both of my kids and, it's something that I did do, but I think that... I wasn't able to do it, in either case, as long as I would have liked since I was too eager to lose the pregnancy weight… which I did and I guess at the time I was... relieved about but, I… do still kind of regret that actually. - … so they would miss out on stuff 'cause, I just couldn't cope with... going out for a big meal with our friends or family, which is really sad. - … there's a lot of time that I've missed out on from them. | 4 |
| **Theme 4: Seeking support** | | |
| 4a. Lack of awareness and information | - It's [being a parent] a topic that never really came up, as I was going through my eating disorder. It was treated as though it was irrelevant, and I don't think it is irrelevant, at all. - …there's certain bits of information you get given 12 times that you go through, you know, in treatment, like the same old things you've read before. I don't think I've ever seen something which is like support for mealtimes with your family, how to navigate conversations or questions from the children, or… you know, I've *never* seen that sort of information. - I felt like when I went to my doctor they kinda didn't really know what to do with me 'cause I wasn't a young, teenage girl. - …I just found the challenges of, you know, I had to take time out of work and obviously I had to parent, whilst trying to recover, as well… and it's not something that you see a lot written about. There's not something that there's a lot of... awareness of I don't think? - … people with eating disorders kind of get pigeonholed as… that it's a young thing… and I think it's important that research shows that it isn't always the case. Even if it starts young, a lot of us continue to struggle much into our adulthood… | 7 |
| 4b. Unique barriers to treatment | - Particularly in the last maybe two years or so, in trying to seek treatment as a parent there's not much that I've been able to find that I'm able to co-ordinate with having a career and children… - When I mention it to anybody, you know about the depression or about the eating disorder, it does worry me that people will think... "She’s not fit to be a parent. It's quite worrying, the things that she's saying", and then all of a sudden there's people knocking on my door saying "You can't be a mother"... That's my biggest concern. - … perhaps treatment of that kind [inpatient]… in the long term is not something that I can do, too often, because of the impact on the family. - …I wouldn't want any sort of authorities to say like “Oh she's, maybe not eating the way she should, like, she can't take care of her kids”… - …where are the support groups for the older people?... because obviously having an eating disorder at an older age brings about different problems. And there doesn't seem to be… that support. | 9 |
| 4c. Support goals | - I think it would just be… an understanding, and a feeling of... acceptance that it's okay to have an eating disorder and be a parent. It's, you know, “you're not gonna be judged for it, we're gonna help you, we'll support you. And we'll support you just like we would support a teenager”. - I think [EXHALES]... the biggest thing would be that sense that you weren't utterly alone and utterly abnormal in your experience. That I wasn't the only parent, with kids, who has an eating disorder. I'm not the only parent with kids that chooses to talk to them about it or, if somebody chooses not to, that they're not alone in that either. I guess it's just that sense of representation and not being an absolute freak of nature and failure as a parent as well. - I think non-judgmental support, would help me… manage my experience better, which would hopefully help me be a better parent. - …because it can get lonely or isolating to be a grown-up… to be a parent and deal with this, and I think the number one benefit would [be] to just be able to talk with people who relate. - I think [my child] would benefit in me accessing support, in that… I'd like to hope that… I would be able to manage it better. - …it's about, being empathetic, and accepting someone and listening to someone non-judgementally, rather than sitting there thinking “God this, you know, this woman, she's [age], she's a mother, and look at the state of her”... - I think it’s getting this piece of mind, and support that when you are dealing with your recovery at the same time you feel like, okay, your kids are doing great as well. | 15 |
| 4d. Practical support suggestions | - I think support from other parents and knowing that you're not alone is always very helpful. That kind of peer support, and you're not weird and you can get better and… eating disorders I guess can be really lonely, and actually having support from other parents who've been through it is so important in parenting full stop. So actually, in the context of an eating disorder I could see how that could be really powerful. - Help for the whole family, 'cause obviously the eating disorder doesn't just affect the one who's got it. It affects the whole family. It affects the other half. It affects the kids. It affects *my* parents… It affects the whole family, so I wish there was a way that the whole family could get support. - I think it's important that… [children] know there's a space for them as well, that they can talk and, yeah that there's support... for *them*, and they don't have to go through it on their own. - … I think it's important that there *is* support for parents. And I think it's important that that's distinguished between support for a young, single person, and a parent with a family. - …I think [support for parents] should be something that's sort of adapted into treatment programs, you know that's there, available for when patients come in that do have children… so that there's something relevant there that they can really… draw from. - …kind of like… a little toolbox of… *this* is information that could help you parenting in this situation, and *this* is information that can help you with the fact that you are experiencing an eating disorder… - …whether it's family counselling or even support groups… where families could be together to… share ideas, share experiences, and have the guidance of a mental health therapist as well as a dietitian. | 15 |
